# Enhanced protein–protein interaction network construction promoted by *in vivo* cross-linking with acid-cleavable click-chemistry enrichment

**DOI:** 10.3389/fchem.2022.994572

**Published:** 2022-11-21

**Authors:** Lili Zhao, Bowen Zhong, Yuxin An, Weijie Zhang, Hang Gao, Xiaodan Zhang, Zhen Liang, Yukui Zhang, Qun Zhao, Lihua Zhang

**Affiliations:** ^1^ CAS Key Laboratory of Separation Science for Analytical Chemistry, National Chromatographic R. & A. Center, Dalian Institute of Chemical Physics, Chinese Academy of Sciences, Dalian, Liaoning, China; ^2^ University of Chinese Academy of Sciences, Beijing, China

**Keywords:** in vivo cross-linking, protein–protein interaction, cross-linked peptide enrichment, click-chemistry reaction, cleavable ligand

## Abstract

Chemical cross-linking coupled with mass spectrometry has emerged as a powerful strategy which enables global profiling of protein interactome with direct interaction interfaces in complex biological systems. The alkyne-tagged enrichable cross-linkers are preferred to improve the coverage of low-abundance cross-linked peptides, combined with click chemistry for biotin conjugation to allow the cross-linked peptide enrichment. However, a systematic evaluation on the efficiency of click approaches (protein-based or peptide-based) and diverse cleavable click-chemistry ligands (acid, reduction, and photo) for cross-linked peptide enrichment and release is lacking. Herein, together with *in vivo* chemical cross-linking by alkyne-tagged cross-linkers, we explored the click-chemistry-based enrichment approaches on protein and peptide levels with three cleavable click-chemistry ligands, respectively. By comparison, the approach of protein-based click-chemistry conjugation with acid-cleavable tags was demonstrated to permit the most cross-linked peptide identification. The advancement of this strategy enhanced the proteome-wide cross-linking analysis, constructing a 5,518-protein–protein-interaction network among 1,871 proteins with widely abundant distribution in cells. Therefore, all these results demonstrated the guideline value of our work for efficient cross-linked peptide enrichment, thus facilitating the in-depth profiling of protein interactome for functional analysis.

## Introduction

Protein–protein interaction is one of the key regulatory mechanisms for controlling protein functions and regulations in various cellular processes (Alberts, 1998). Nowadays, many technologies have been developed to globally study protein–protein interactions (PPIs), especially in a cellular context (Titeca et al., 2019), such as affinity purification-mass spectrometry (AP-MS) and proximity-labeling techniques. These methods make use of overexpressed tagged bait proteins, which might introduce artifactual interactors to the bait protein and thus lead to false-positive results. Recently, chemical cross-linking coupled with mass spectrometry (CXMS) has become a powerful method for PPI analysis with the advantage of locating the interface between interacting proteins. This strategy has been successfully employed for unraveling the protein complex topology and protein–protein interacting interfaces on a proteome-wide level, especially in native cells (Chavez et al., 2019; Gotze et al., 2019; Wheat et al., 2021).

In the CXMS strategy, a cross-linker is used to covalently link the active groups of amino acid residues positioned in close proximity between and within proteins. Since the cross-linkers primarily react with the amino acids on the protein surface, it greatly limits the yield of cross-linking products. Exemplified by N-hydroxysuccinimidyl (NHS) ester reactive cross-linkers, which target lysine residues of the highest abundance on the protein surface, multiple types of peptide mixtures exist in the digested products, including regular, mono-linked, loop-linked, and cross-linked peptides. Among the peptide mixture, cross-linked peptides are the most imformative species for protein interactions but with the least abundance (Yu and Huang, 2018; Iacobucci et al., 2019; Leitner et al., 2020). Thus, the analysis of the low-abundance cross-linked peptides was seriously inhibited by the non-cross-linked peptides. In response, many efforts have been made to increase the relative abundance of cross-linked peptides (Leitner et al., 2012; Tan et al., 2016; Petraryl et al., 2019; Steigenberger et al., 2019; Wheat et al., 2021). Among the reported methods, enrichable cross-linkers incorporating an affinity handle were the most promising. With the superiority of a small steric hindrance in facilitating the cross-linker transported into the cell for *in vivo* cross-linking, alkyne/azide-tagged cross-linkers were increasingly used by introducing biotin with click chemistry, followed by streptavidin bead purification (Kaake et al., 2014; Rey et al., 2021; Wheat et al., 2021). Taking advantage of this strategy, Wheat et al. identified 13,904 unique lysine–lysine linkages from *in vivo* cross-linked HEK 293 cells by peptide-based click chemistry, permitting the construction of 5,401 PPIs, which is the largest *in vivo* PPI network to date (Wheat et al., 2021).

However, the systematic evaluation on the effect of different click approaches and cleavable types for the cross-linked peptide enrichment and release is ambiguous. Both protein-based (Kaake et al., 2014; Zhao et al., 2022) and peptide-based (Rey et al., 2021; Wheat et al., 2021) click chemistry have been used for cross-linked peptide enrichment. Meanwhile, with the features of bio-orthogonality, quick reaction speed, and great specificity, the click-chemistry reaction also has been successfully applied in some other studies, such as activity-based protein profiling, enzyme-inhibitors screening, and protein labeling in proteomic analysis (Hahne et al., 2013; Peng et al., 2016; Yang et al., 2018; Mcconnell et al., 2020; Yao et al., 2021). Lately, as to profiling of low-abundance nascent proteome, peptide-based click chemistry compared to the conventional protein level has shown a 2-fold increase in AHA-containing peptide identification with potentially reducing steric hindrances (Sun et al., 2020). Moreover, Li et al. (2022) evaluated the performance of five cleavable biotin tags in the three most common chemoproteomic workflows for cystine site identification to provide a practical guidance for a peptide-centric chemoproteomic study. Thus, it is extremely necessary to investigate the effect on different combinations of click-chemistry and cleavage modes for cross-linked peptide identification.

In this work, we evaluated the efficiency of alkyne-tagged cross-linkers with three types of cleavable azide–biotin ligands conjugated on both protein- and peptide-based click chemistry, respectively, for cross-linked peptide enrichment. The strategy presented here could provide technological guidance for click-chemistry-based cross-linking enrichment, allowing in-depth PPI analysis for charting protein interaction landscapes in cells.

## Results and discussion

### Enrichment of cross-linked peptides with different click-chemistry approaches

**FIGURE 1:**
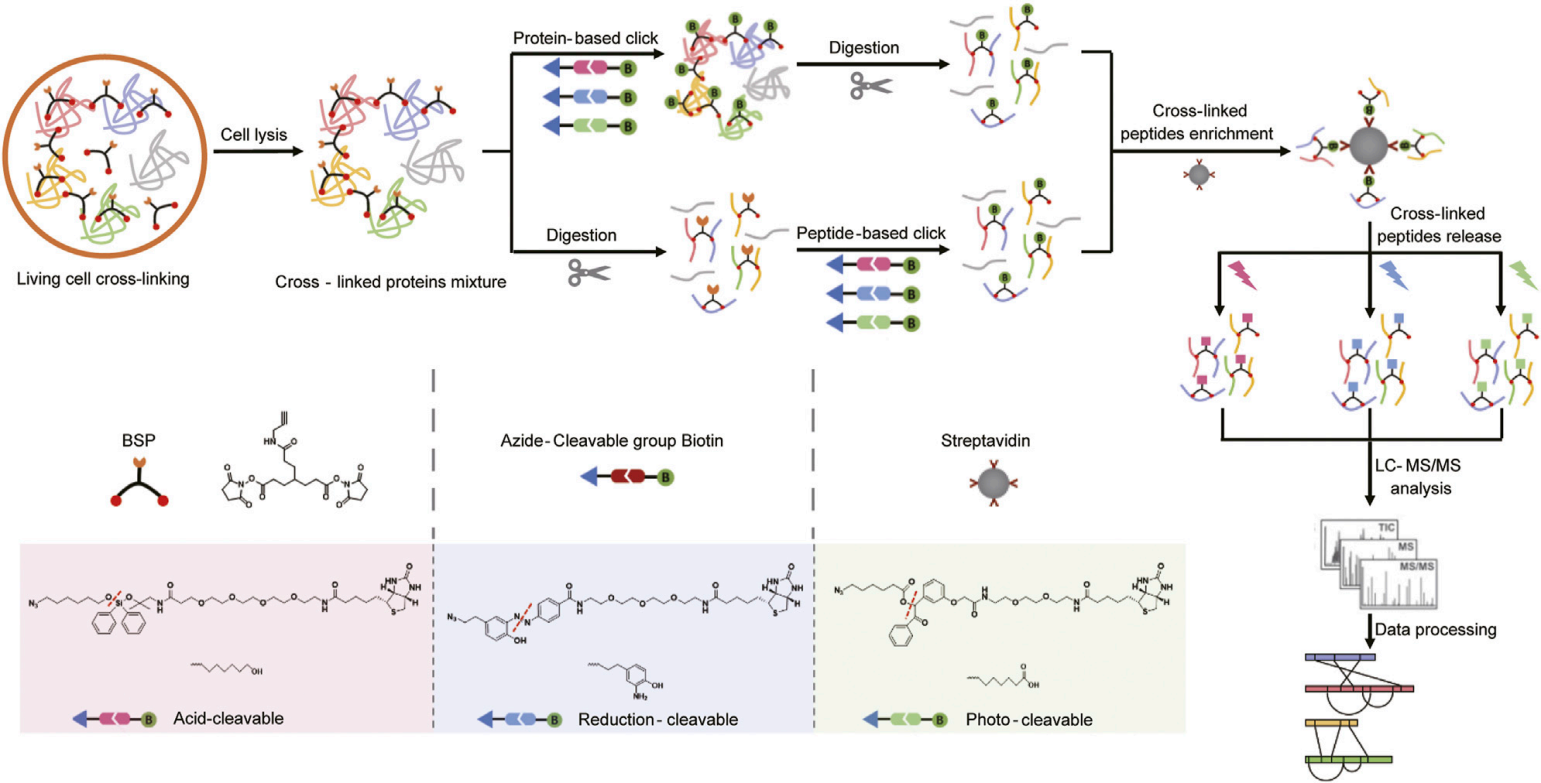
Flow diagrams of both protein-based and peptide-based click-chemistry reactions for *in vivo* cross-link analysis. Bottom left: chemical structures of the cross-linker BSP, three cleavable azide–biotin reagents, and the tags modified to the cross-linker after cleavage are, respectively, illustrated, in which red dashed lines represent the cleavable sites.

Increasing the coverage of chemical cross-linking remains a great challenge due to the low-abundance of cross-linked peptides. As shown in Figure 1, *in vivo* cross-linking was performed with our previously developed cross-linker bis (succinimidyl) with a propargyl tag (BSP) (Gao et al., 2022) (Supplementary Figure S1), which is membrane permeable and consists of a homobifunctional NHS ester reactive group and an alkyne enrichable tag for living-cell cross-linking in minutes. The small size and specific reactivity made the alkyne group a preferred tag by introducing biotin *via* click chemistry for cross-linked peptide enrichment. Specifically, we introduced the biotin group before (protein-based) and after (peptide-based) proteolysis, followed by the enrichment of biotin-labeled cross-linked peptides using streptavidin beads. Subsequently, to avoid the hydrophobicity of biotin interfering with LC separation, three cleavable azide–biotin ligands of acid-, reduction-, and photo-cleavable specificity with diverse solubility and size were investigated (Supplementary Figure S2). The acid-cleavable ligand has the longest chain and best hydrophilicity, but the whole process needs to avoid acids. The reduction-cleavable ligand has a bright yellow color from the azobenzene group; thus, the binding between cross-links and the ligands could be visualized, while turning white after cleavage. But the reduction-cleavable reaction occurs in a high concentration of salt; thus, the desalting step is necessary. In addition, the inconvenient dark environment needs to be maintained during the whole process for photo-cleavable ligands. For the acid- and photo-cleavable ligand-generated samples, the desalting step is not needed in theory, which is more MS-compatible and causes less loss. In addition, considering the lower sample complexity at the protein level and small steric hindrance at the peptide level for click-chemistry reaction, it is also necessary to investigate the effects of three cleavable click-chemistry ligands for cross-link enrichment and release, respectively.

### Evaluation of the cross-linked peptide enrichment efficiency

**FIGURE 2:**
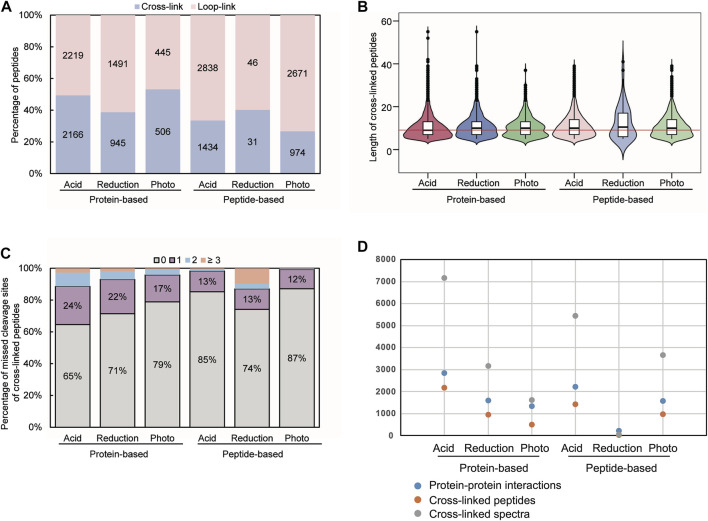
Comparison of **(A)** type proportion, **(B)** length, and **(C)** missed cleavage of the cross-linked peptides, as well as **(D)** number of cross-linked spectra, cross-linked peptides, and protein–protein interactions.

To evaluate the cross-linked peptide enrichment efficiency of the approaches with three cleavable ligands on protein and peptide-based click chemistry, we compared the type, identification number, length, and missed cleavage of the cross-linked peptides. First, a notably higher proportion of cross-linked peptides was identified using protein-based click chemistry than that with the peptide-based method (Figure 2A). This might be due to the relatively higher sample complexity for the click chemistry labeling of alkyne tags in cross-links at the peptide level than that of the protein-based approach. In addition, other than the equivalent chance of the click-chemistry reaction for cross-links and loop-links at the peptide level, bigger steric hindrance existed for cross-linked peptides than loop-linked peptides, compromising the chance of cross-linked peptides to encounter click-chemistry ligands. Importantly, we found the residual biotin had a fatal defect on mass spectrometry detection in our previous work (Gao et al., Anal. Chem. 2022). Therefore, in this work, we made biotin carefully removed by acetone precipitation method in protein-based approach, while for peptide-based click reaction, we adopted the same method from the previously delicate work (Fu et al., Nat Protoc. 2020) without SCX treatment for recovery improvement. Expectedly, the identification number of cross-linked peptides in both protein and peptide based approaches were obviously increased. Among the six conditions, protein-based click-chemistry conjugations with acid-cleavable tags identified the most cross-linked peptides, followed by peptide-based click-chemistry conjugations with acid-cleavable tags. It might be attributed to the flexible spacer arm, good hydrophilicity, and small reaction steric hindrance of the acid-cleavable azide–biotin reagent. In contrast, the approach of photo-cleavable ligand labeling by click chemistry at the protein level identified the least. Meanwhile, the inconvenient experimental condition of darkness is required for a longer time. Given peptide-based click-chemistry conjugations with reduction-cleavable tags identified the least cross-linked peptides, which might be due to the larger local hydrophobicity and steric resistance of azide–biotin reagents, this result was not discussed in the subsequent comparison. Furthermore, we compared the length of the cross-linked peptides, which could influence chromatographic separation and MS identification. The length of the cross-linked peptides was in mere difference (Figure 2B). However, the missed cleavage of the cross-linked peptides on protein-based click chemistry was slightly higher than that on peptide-based click chemistry (Figure 2C), which can be mainly attributed to the low efficiency of enzymatic hydrolysis of proteins due to the steric hindrance of azide–biotin ligands. Then, we totally compared the number of cross-linked spectra, cross-linked peptides, and PPIs of these methods. The acid-cleavable tag in combination with protein-based click chemistry outperforms all other methods (Figure 2D). The average number of cross-linked sites for PPIs identified in protein-based methods was more than that in peptide-based methods. Taken together, the protein-based click chemistry conjugated with the acid-cleavable azide–biotin ligand approach was recommended for cross-linked peptides enrichment to realize the in-depth protein interactome identification. Detailed identification results are listed in Supplementary Material S1.

### In-depth profiling of human cell cross-linking protein interactome

**FIGURE 3:**
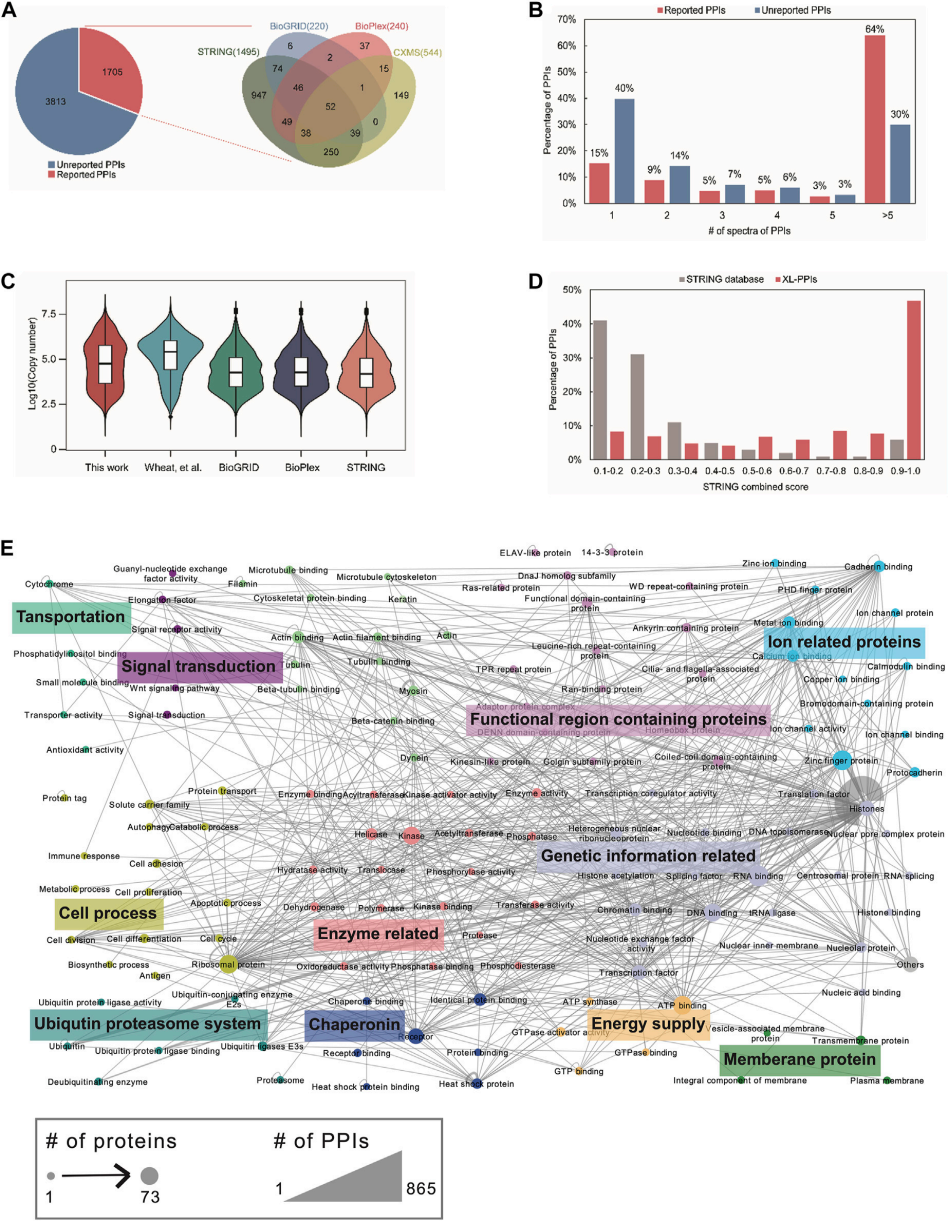
Protein–protein interaction analysis. **(A)** Overlap of our identified PPIs with the existing PPI databases of STRING, BioGRID, BioPlex, and referenced cross-linking databases. **(B)** Distribution of the number of spectra between reported PPIs and unreported PPIs. **(C)** Copy number distribution of the interaction proteins among different protein interaction databases. Protein copy numbers are from the study of previous study (Bekker-Jensen et al., 2017). **(D)** Comparison of STRING-score distribution between the identified PPIs and all the human PPIs in the STRING database. **(E)** Protein interaction network mapped by the identified cross-linked proteins. The nodes are color coded based on protein functions. The size of the nodes is proportional to the number of proteins included in the corresponding classification. Thickness of the lines represents the number of protein interactions.

To enhance the identification coverage of cross-linking, the cross-linked peptides generated from protein-based click-chemistry conjugation with acid-cleavable tags were further fractionated by high-pH RPLC and identified by low-pH nanoRPLC-ESI-MS/MS analysis. In total, 30,499 inter-protein linkages of 5,518 PPIs involved in 1,871 proteins were identified. Detailed identification results are provided in Supplementary Material S2. By matching with the existing PPI databases (Figure 3A and Supplementary Material S3), 1,705 PPIs were reported, and 3,813 PPIs were newly identified, which could most likely be attributed to the capture bias and distinct filter threshold of these PPI profiling methods, as well as cell type and cell state heterogeneity. Further gene ontology analysis for unreported PPIs revealed they are closely related to protein translation, synthesis, and folding (Supplementary Figure S3), which are usually difficult to resolve by *in vitro* approaches. In addition, we estimated the protein abundance of our XL–PPI proteomes with the aforementioned compared PPI databases (Figure 3C). A wide abundance distribution was obtained in our dataset. Although the protein abundance distribution from the integrated databases of BioGRID, BioPlex, and STRING was higher than that of the cross-linking method, our identified cross-linked proteins were relatively less abundant compared to Wheat et al.’s data obtained by click-chemistry labeling of alkyne tags at the peptide level (Wheat et al., 2021). This result also confirmed the higher enrichment efficiency of click chemistry performed at the protein level. Moreover, we investigated the accuracy of the PPIs on account of STRING database. Among 5,518 PPIs, 1,495 were covered in the STRING database and matched to the corresponding reliability score. Among them, 47% (699) of the PPIs were in the highest score range of 0.9–1.0, while more than 72% of the whole human PPIs collected in the STRING database were in the relatively low score range of 0.1–0.3 (Figure 3D), indicating good reliability of our cross-linking data. Furthermore, by counting the spectrum number distribution of PPIs (Figure 3B), more than 76% of the reported PPIs were assigned more than two spectra. For evaluating the confidence of unreported PPIs, we used bioinformatic methods to calculate the GO correlation of PPIs (Yu, 2020). We applied this method to compare the similarities of molecular functions (MFs) of the reported PPIs in the STRING database (100,000 PPIs were randomly selected) and unreported PPIs in our data (Supplementary Material S4). The distribution of MF correlation coefficients was similar as reported PPIs and unreported PPIs, which implies that most of our identified unreported PPIs were as reliable as those in the STRING database from this comparison. To enhance the PPI confidence, three spectra was set as a cut-off to filter the unreported PPIs, while all reported PPIs identified was kept.

Finally, to visualize the correlation of the filtered PPIs, the interactome network was profiled and classified according to the protein function into 134 subgroups, mainly associated with transcription and translation, protein binding, signal transduction, and cell metabolism (Figure 3F). Detailed interaction protein classification is provided in Supplementary Material S4.

### Spatial analysis of protein–protein interactions

**FIGURE 4:**
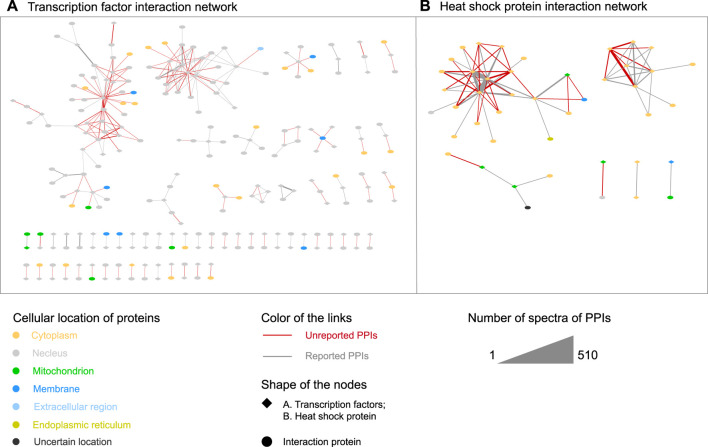
**(A)** Transcription factors and **(B)** heat-shock protein interaction networks from our identified cross-linking PPIs.

*In vivo* cross-linking could tackle the limitation of AP-MS for capturing weakly bound protein complexes, avoiding the loss of spatial information and providing direct protein interactions. Exploring the spatial natures of protein complexes is crucial to the precise regulation of critical phenotypic outputs. Transcription factors (TFs) and heat shock proteins (HSPs) (Figure 4), which are hardly detectable by *in vitro* methods, were exemplified. Spatial dynamics of PPIs might be explained by multiple localizations of proteins within cells.

TFs are considered relatively low abundance in the proteome, regulating almost every aspect of life, ranging from embryonic development to carcinogenesis. The resource of TF regulators will have implications for our understanding of how TFs execute their regulatory functions. We identified 251 TF-containing PPIs consisting of 106 TF and 135 TF interacting proteins (Figure 4A). Among the PPIs, 109 were reported and the 142 unreported PPIs could effectively complement the current network. The TF interaction proteins were mainly located in the nucleus to regulate the highly dynamic transcription and signal transduction processes. There were also a small number of proteins located in non-nucleus locations to regulate specific functions. The reason of our method to allow the identification of the relatively low-abundance PPIs with transient interactions was mainly attributed to the unique microreactor feature provided by the intracellular crowding and confinement environment. This feature was beneficial to the occurrence of cross-linking reactions, especially crucial for low-abundance proteins, which was evidently in contrast with the dispersal of proteins in cell lysate. Detailed protein interaction networks and cellular locations were provided in Supplementary Material S5.

HSPs are highly conserved and dynamic/variable molecular chaperones for protein folding, transport degradation, and translocation to maintain protein homeostasis. In total, we identified 78 HSP interactions consisting of 23 HSP and 21 HSP interacting proteins (Figure 4B). Among the PPIs, 44 were reported and the 34 unreported PPIs could effectively complement the current network. The HSP interaction proteins were mainly enriched in the cytoplasm. Considering the weak and dynamic binding features of the chaperone proteins in the signal transduction, our method might be helpful to understand the regulatory mechanisms of chaperone mediation for intracellular protein homeostasis. Detailed protein interaction networks and cellular locations are provided in Supplementary Material S6.

## Conclusion

A systematic study on the effects of different click chemistry approaches and cleavable ligands for the cross-linked peptide enrichment and identification was performed. The strategy of protein-based click-chemistry conjugations with acid-cleavable ligands was proven to generate the most cross-linked peptides. With the advancement of this strategy, the efficiency of click-chemistry-based cross-linking enrichment was enhanced, and an in-depth profiling of human protein interactome with 30,499 inter-protein linkages of 5,518 PPIs involved in 1,871 proteins was constructed with *in vivo* cross-linking. Therefore, all these results demonstrated the great promise of our work for proteome-wide mapping of protein interaction landscapes in cells.

## Materials and methods

### Cell culture

A total of 293T cells were maintained in DMEM (Gibco, Life) and supplemented with 5% FBS (Premium, South America) and antibiotics (100 IU ml^−1^ penicillin and 100 μg ml^−1^ streptomycin) at 37°C under 5% CO_2_ atmosphere.

### *In vivo* cross-linking

The cells were harvested and washed 3 times with 1× PBS before cross-linking in centrifuge tubes. The cell pellet of 2 × 10^7^ cells in each group was resuspended and cross-linked in 1.2 ml 1× PBS (1% DMSO, v/v) with 5 mM of BSP (Gao et al., 2022) at room temperature for 5 min.

### Protein-based click chemistry

(1) The cross-linked cells were collected and 0.2% SDS was added (1× PBS) to extract protein. (2) Click chemistry was performed by adding cleavable azide–biotin reagents, THPTA, CuSO_4_, and sodium ascorbate to the protein sample with the molar concentration ratio to the cross-linker of 1:10, 4:10, 0.5:10, and 1.25:10, respectively. The volume of the reaction was 2.5 ml. The resulting mixture was rotated at 60°C for 2 h (Supplementary Table S1). Then, the proteins were deposited by acetone precipitation. (3) The precipitated protein pellets were air dried and resuspended in 8 M urea (50 mM NH_4_HCO_3_), following by reduction (8 mM DTT, 25°C, 1 h) and alkylation (32 mM IAA, 25°C, 30 min, dark), and the samples were diluted to 1 M urea with 50 mM NH_4_HCO_3_ and digested with trypsin at 37°C overnight. The experimental procedure was consistent with our previous mature works (Gao et al., 2022; Zhao et al., 2022).

### Peptide-based click chemistry

(1) The cross-linked cells were collected and lysis buffer was added [50 mM HEPES, 150 mM NaCl, pH 7.6 with 1% protease inhibitor cocktail purchased from Thermo Fisher Scientific (Waltham, MA, United States)] to extract protein, followed by reduction (8 mM DTT, 25°C, 1 h) and alkylation (32 mM IAA, 25°C, 30 min, dark). Then, the proteins were deposited by methanol–chloroform precipitation. The precipitated protein pellets were air dried and resuspended in 50 mM NH_4_HCO_3_ and digested with trypsin at 37°C overnight. Next, HLB SPE cartridges were used to efficiently separate peptides from inorganic salts under neutral conditions. (2) Click chemistry was performed by adding cleavable azide–biotin reagents, TBTA, CuSO_4_, and sodium ascorbate to dried peptide digests to the final concentration of 1, 1.25, 10, and 10 mM, respectively (Supplementary Table S1). The volume of the reaction was 80 μl. The resulting mixture was rotated at room temperature for 2 h. Then, SCX was used for cleaning excess click-chemistry reagents. The experimental procedure was consistent with the previous mature work (Fu et al., 2020; Li et al., 2022).

### Cross-linked peptide enrichment

The resulting peptide mixture was incubated with streptavidin beads for 2 h at room temperature. Streptavidin-bound peptides were washed extensively before cross-linked peptide release.

### Cross-linked peptide release

For the acid-cleavable reagent (DADPS Biotin–Azide, CLICK CHEMISTRY TOOLS)-ligated sample, streptavidin-bound peptides were eluted using 10% of formic acid (FA) three times at room temperature. For the reduction-cleavable reagent (Azo Biotin–Azide, Sigma-Aldrich)-ligated sample, streptavidin-bound peptides were eluted using 300 mM Na_2_S_2_O_4_ in 20 mM HEPES, 6 M urea, and 2 M thiourea buffer, pH 7.6, at room temperature. For the photo-cleavable reagent (UV Cleavable Biotin–Azide, Kerafast)-ligated sample, streptavidin-bound peptides were eluted by exposing under 365 nm UV light for 1 h at room temperature. The peptides were collected and desalted with home-made C18 Tips.

Before peptide release, the photo-cleavable reagent labeled sample should be performed with light protection, and the acid-cleavable reagent labeled sample should be performed avoiding acid and not doing the SCX procedure.

### LC–MS/MS analysis

LC-MS/MS was performed using an Orbitrap Fusion Lumos mass spectrometer (Thermo Fisher Scientific) coupled with an Easy-nLC 1,200 system. A flow rate of 600 nL min^−1^ was used, where mobile phase A was 0.1% FA in H_2_O, and mobile phase B was 0.1% FA in 80% ACN and 20% H_2_O. Peptides were directly injected into the analytical column, prepared in-house, with an internal diameter of 150 μm packed with ReproSil-Pur C18-AQ particles (1.9 μm, 120 Å, Dr. Maisch) to a length of approximately 30 cm. Mass spectrometry was operated in a data-dependent mode with one full MS scan over the m/z range from 350 to 1,500, MS scan at R = 60,000 (m/z = 200), followed by MS/MS scans at R = 15,000 (m/z = 200), and RF Lens (%) = 30, with an isolation width of 1.6 m/z. MS^1^ acquisition was performed with a cycle time of 3 s. The AGC target for the MS^1^ and MS^2^ scan were 400,000 and 50,000, respectively, and the maximum injection time for MS^1^ and MS^2^ were 50 ms and 30 ms, respectively. The precursors with charge states +3 to +7 with intensity higher than 20,000 were selected for HCD fragmentation, and the dynamic exclusion was set to 40 s. Other important parameters were default charge, +2 and collision energy, 30%.

### Data processing

pLink 2 (Chen et al., 2019) software (version 2.3.11) was used for cross-link identification, and the results were filtered by applying separate 1% FDR control of intra-protein and inter-protein results at the spectral level. The search parameters used are as follows: instrument, HCD; precursor mass tolerance, 20 ppm; fragment mass tolerance, 20 ppm, the peptide length was set to 5–60, carbamidomethyl [C] as fixed modification, and acetyl [protein N-term] and oxidation [M] as variable modification. Cross-linker was set as BSP-acid-cleave (cross-linking sites K and protein N terminus, cross-link mass-shift 376.211, mono-link mass-shift 394.222), BSP-reduction-cleave (cross-linking sites K and protein N terminus, cross-link mass-shift 411.191, mono-link mass-shift 429.201), and BSP-photo-cleave (cross-linking sites K and protein N terminus, cross-link mass-shift 390.190, mono-link mass-shift 408.201). Trypsin as the protease with a maximum of three missed cleavages was allowed. The database on *Homo sapiens* was downloaded from UniProt on 2022-03-29.

## Data Availability

The mass spectrometry data have been deposited in jPOST repository (PXD035355) and links of the data was http://proteomecentral.proteomexchange.org/cgi/GetDataset?ID=PXD035355.
