# Supplementary material for: Enhanced protein–protein interaction network construction promoted by in vivo cross-linking with acid-cleavable click-chemistry enrichment
Source: Front Chem. 2022 Nov 21;10:994572. doi: 10.3389/fchem.2022.994572 (PMC9720147; doi:10.3389/fchem.2022.994572)
Supplement: Supplementary file 4 [file DataSheet1.PDF]

Supplementary Material

1 Supplementary Tables and Figures

1.1 Supplementary Tables

Reaction format	System	Cleavable azide-biotin reagent	$CuSO_4$	VC	Ligand	Reaction temperature	Time
Protein	0.2% SDS (1×PBS), 2.5 mL	20 mM, 30 μL	20 mM, 15 μL	50 mM, 15 μL	160 mM THPTA, 15 μL	60 ℃	2 h
Peptide	40 μL H_2O + 20 μL ACN	40 mM, 2 μL	100 mM, 8 μL	100 mM, 8 μL	50 mM TBTA, 2 μL	25 ℃	2 h

Supplementary Table 1. Click chemistry reaction conditions.

1.2 Supplementary Figures

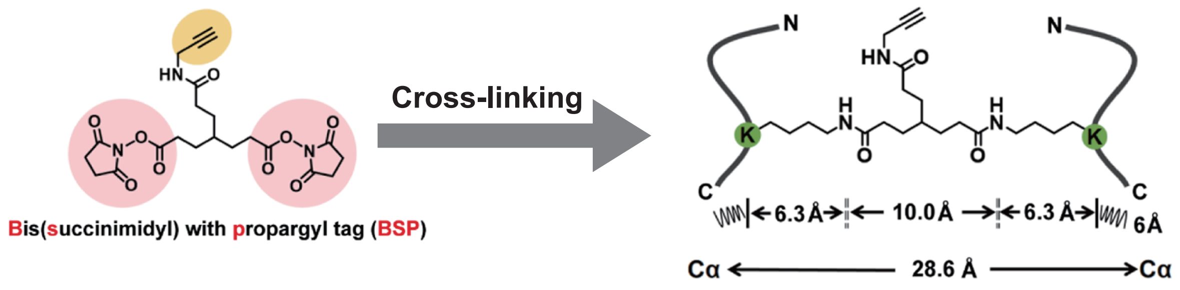

Supplementary Figure 1. The Chemical structure of BSP and schematic diagram of cross-linking sites in proteins

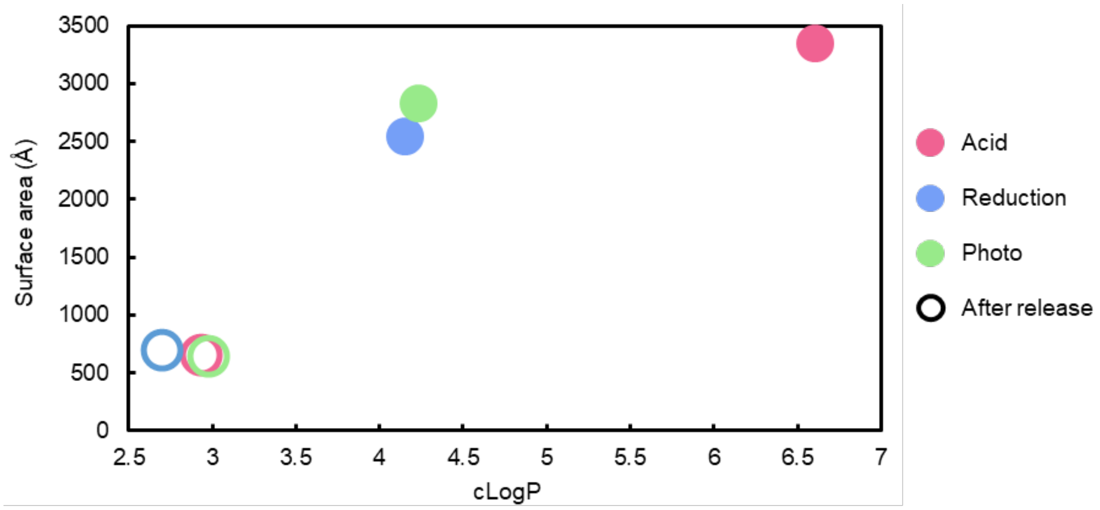

Supplementary Figure 2. Basic properties of the three cleavable azide-biotin reagents. The clog P value (horizontal axis) is a measure for hydrophilicity, which is obtained by ChemDraw Professional 19.0. The smaller the value, the better hydrophilicity the crosslinker. Surface area (vertical axis) is accomplished by PyMol software to investigate the structural space of a crosslinker.

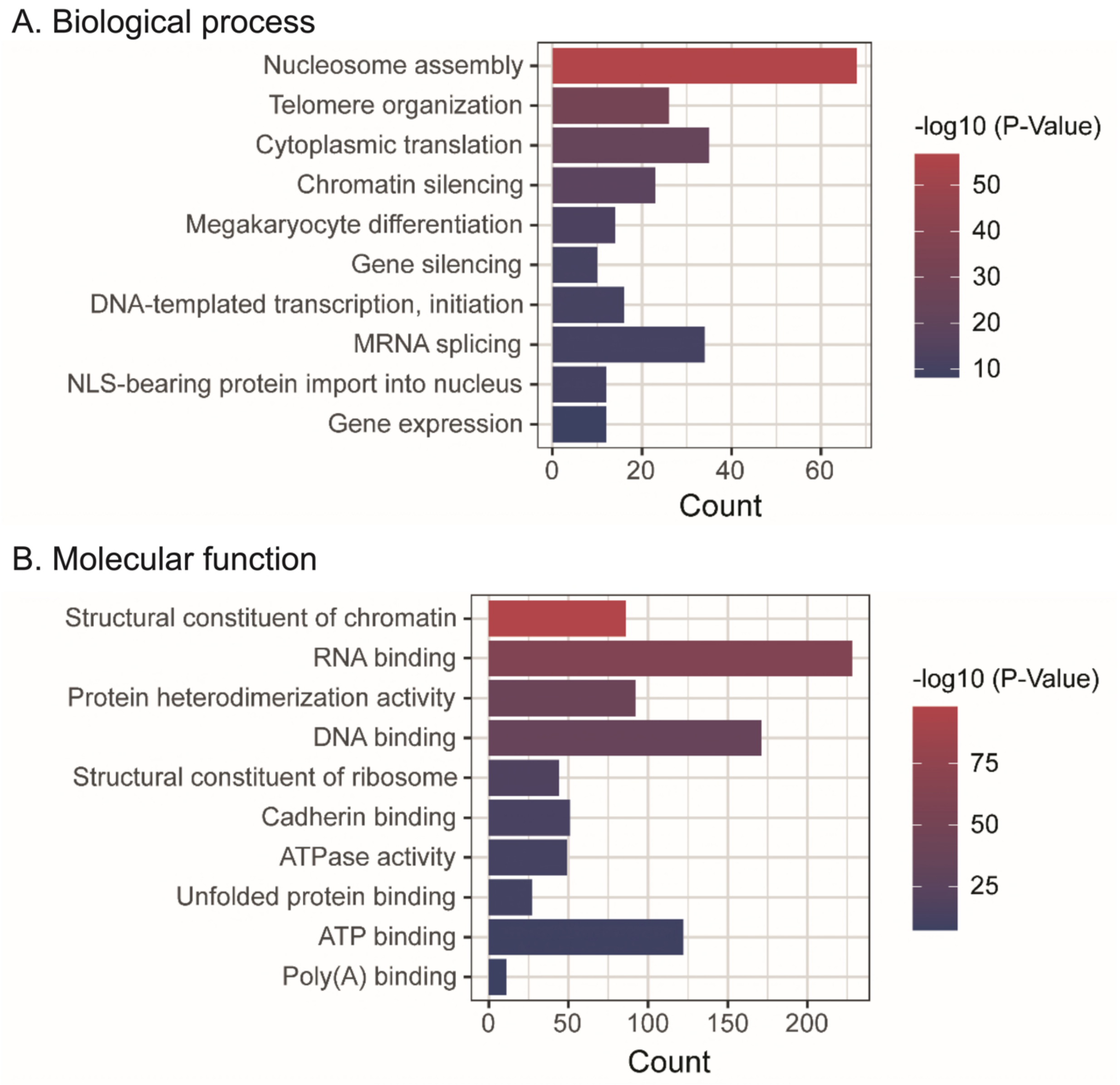

Supplementary Figure 3. Biological process **(A)** and molecular function **(B)** of the unreported PPIs related proteins.

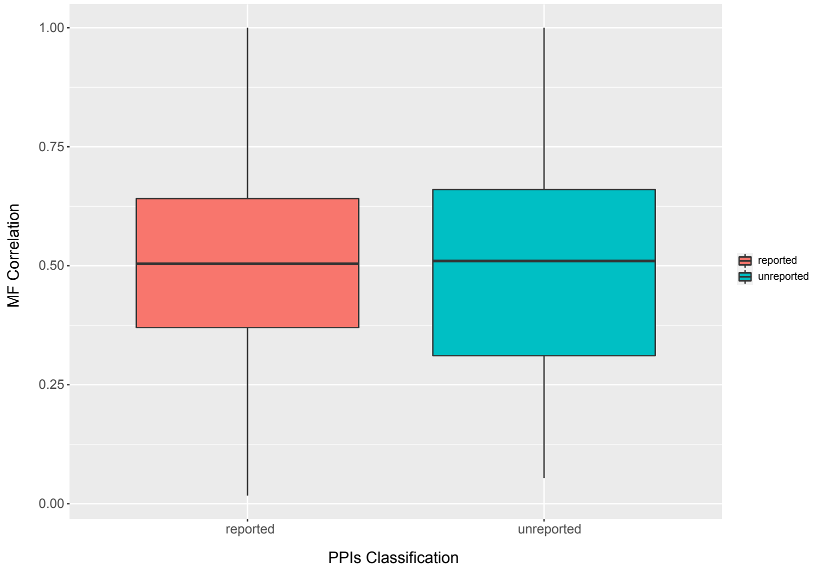

Supplementary Figure 4. Comparison on MF correlation of the reported PPIs in String database and unreported PPIs from more than 2 cross-linked spectra in our data.

2 Supplementary Datasets

Supplementary Data 1. Cross-linking results of in vivo cross-linking combined with two types of click chemistry reaction and three kinds of cleavable azide-biotin reagents.

Supplementary Data 2. Cross-linking results of in vivo cross-linking combined with protein-based click chemistry reaction and the acid-cleavable reagent.

Supplementary Data 3. The identified protein interactions reported in STRING, BioGRID, BioPlex and the cross-linking database.

Supplementary Data 4. Function annotation of the interaction proteins.

Supplementary Data 5. Transcription factor protein interaction network and cellular location of the interaction proteins.

Supplementary Data 6. Heat shock protein interaction network and cellular location of the interaction proteins.
